# Genotyping microsatellites in next-generation sequencing data

**DOI:** 10.1186/1471-2105-16-S2-A5

**Published:** 2015-01-28

**Authors:** Harriet Dashnow, Susan Tan, Debjani Das, Simon Easteal, Alicia Oshlack

**Affiliations:** 1 Life Science Computation Centre, Victorian Life Sciences Computation Initiative, Carlton, VIC, Australia; 2 The University of Melbourne, Parkville, VIC, Australia; 3 Murdoch Childrens Research Institute, Parkville, VIC, Australia; 4 John Curtin School of Medical Research - Australian National University, Canberra, ACT, Australia

## Background

Microsatellites are short (2-6bp) DNA sequences repeated in tandem, which make up approximately 3% of the human genome [1]. These loci are prone to frequent mutations and high polymorphism with the estimated mutation rates of 10^−2^ - 10^−6^ events per locus per generation, orders of magnitude higher than other parts of the genome [2]. Dozens of neurological and developmental disorders have been attributed to microsatellite expansions [3]. Microsatellites have also been implicated in a range of functions such as DNA replication and repair, chromatin organisation and regulation of gene expression [4].

Traditionally, microsatellite variation has been measured using capillary gel electrophoresis [5]. In addition to being time-consuming, and expensive, this method fails to reveal the full complexity at these loci because it does not directly sequence the fragment but only measure the number of bases in the repeat.

Next-generation sequencing has the potential to address these problems. However, determining microsatellite lengths using next-generation sequencing data is difficult. In particular, polymerase slippage during PCR amplification introduces stutter noise. A small number of software tools have been written to genotype simple microsatellites in next-generation sequencing data [6-8], however they fail to address the issues of SNPs and compound repeats, and in some cases provide only approximate genotypes.

We have begun to develop a microsatellite genotyping algorithm that addresses these issues, providing high accuracy as well as more detailed analysis of microsatellite loci. We have validated it using high depth amplicon sequencing data of microsatellites near the *AVPR1A *gene.

## Results

**Figure 1:**
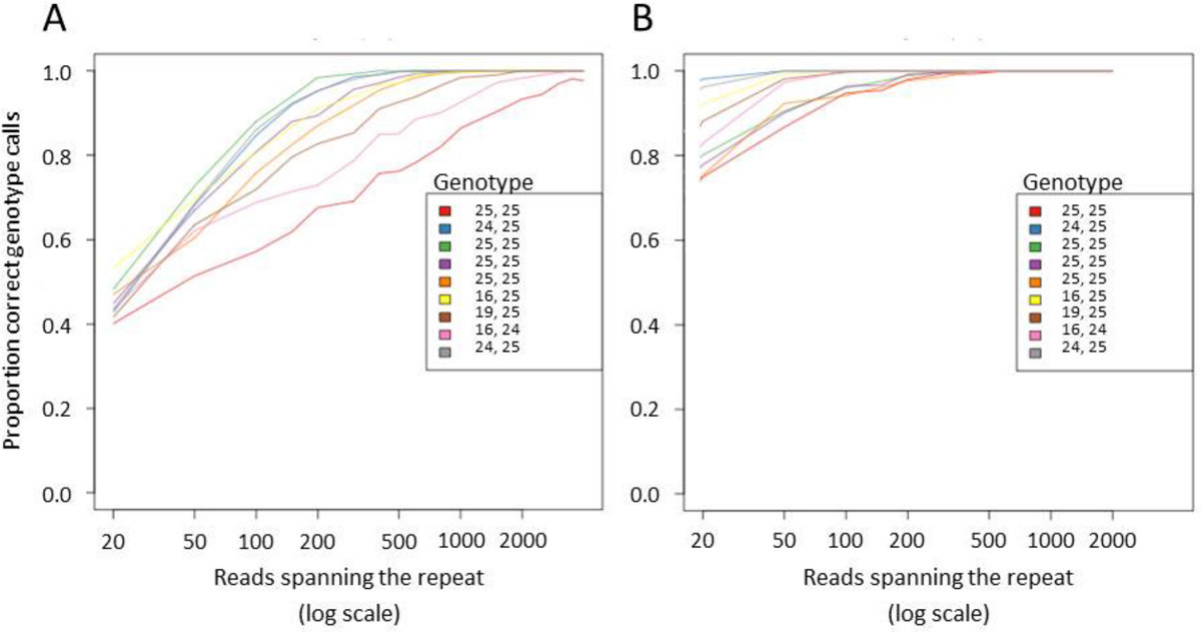
**Genotyping accuracy at the (AC)_n _promoter locus as a function of the number of reads spanning the microsatellite**. 20 to 3000 reads were sampled with replacement from those spanning the microsatellite. This was done 1000 times for each depth. A shows the portion of genotypes that were exactly correct, B shows the proportion of genotypes that were correct to within one repeat unit.

We found high concordance between our algorithm and repeat lengths obtained by electrophoresis, manual inspection and Mendelian inheritance (Table 1). By subsampling the reads, we found that our model is accurate to within one repeat unit down to coverages that we would expect in standard exome sequencing (Figure 1). Additionally, we detected polymorphic single nucleotide changes within some microsatellites.

**Table 1:** Concordance of microsatellite variance calls three validation methods: electrophoresis, manual inspection and Mendelian inheritance.

Validation method	Concordant #	Concordant %
Electrophoresis	9/9	100%

Manual inspection	17/18	~95%

Mendelian inheritance	18/18	100%

## Conclusions

The algorithm was approximately 95% correct at calling the exact same genotype on high depth sequencing data. When it did call a genotype incorrectly, the genotype was only one repeat unit different. The algorithm can perform at approximately 90% accuracy to within one repeat unit with as few as 20 informative reads and reaches almost 100% accuracy to within one repeat unit with 100 or more informative reads.

Future work will include expanding the algorithm to genotype compound microsatellites and further validation and comparison with other algorithms will be performed on whole genome data sets.
